# Everybody's Page

**Published:** 1889-03-09

**Authors:** 


### Everybody's Page.

Cholera is raging in Tien-Tien, in China. The mortality caused by it is said to be 60 per cent.

Dr. B. G. Pullin states that warts rapidly disappear if arsenic is administered internally.

An itinerant dispensary has been established in Roumania, where doctors are scarce. Last summer nine thousand patients were treated by it.

Hippocrates, four and a-half centuries before the Christian era, divided the life of man into seven ages. Had Shakespeare ever heard of this when he wrote his oft-quoted description?

Paul Bert, whose scientific enthusiasm more than bordered on recklessness, tested the value of vaccination in a courageous manner. He had himself vaccinated, and afterwards was inoculated, at Havre, with virus from a man who was dying of small-pox. He did not contract the disease.

Typhoid-fever patients feel better after a salt water bath than a fresh one, and the salt baths reduce the temperature, pulse, and respiration more; but the effect on the two last is not so great as to indicate any very powerful influence on the functional activity of heart and lungs.

A new, and apparently very successful, method of preserving butter consists in adding to it a very small portion of salicylic acid dissolved in two parts of lactic acid, and ninety-eight parts of water. How little salicylic acid is required to keep butter fresh for an indefinite length of time may be calculated from the fact that not m ore than one grain is employed for every hundred kegs of butter.

A new use has been discovered for the poppy. Once sown it is self-perpetuating, and is indeed almost impossible to get rid of. It forms a network of roots that cannot be exterminated without great difficulty, and it is therefore admirable for keeping embankments in place. Within the last two or three years eminent French engineers have undertaken the sowing of railway embankments with poppies, with a view to prevent their being destroyed by heavy rains, or by upheaval when the frost is breaking up in the spring.

In 1882, a judge, who had been troubled by the often conflicting evidence of experts of various kinds, said there were three classes of liars: 1st, liars,; 2nd, great liars; 3rd, scientific witnesses. On the other hand, these scientific witnesses often complain, as Christison did, that the examining counsel either do not let them tell the whole evidence at their command, but only so much as suits the aspect of the case under trial, or else try to lure them into hypotheses and inference beyond what scientific accuracy justifies.

Dr. Lichtenbag, of Buda-Pesth, says that out of 250 railway employés whom he examined, 92, or more than a third, suffered from ear disease. Engineers are especially liable to rheumatism and pneumonia, and after some years' service a certain proportion of them become dull of sight and hearing. Others suffer from a mild form of spinal concussion, muscular feebleness, and continuous pains in the limbs. They are also apt to develop a peculiar mental state, a sort of cerebral irritation, with excessive nervousness and morbid sensations of fear. The life of railway men does not seem to be a healthy one. Duchesne says that for the first few years the men improve in health, but at the end of ten years they are tired out; in fifteen they are actual sufferers; and very few can remain in the service after twenty.

One fourth of the deaths in Paris are those of children under two years of age.

Tea-drinking is increasing in France, the quantity consumed having doubled within the last thirty years.

Mollin is a new vehicle for the application of drugs to the skin. It is a soap containing glycerine and an excess of fat, and is said to be easily absorbed.

It is said that convulsions may frequently be cut short by turning the patient on his left side. Nausea, occurring as an after-effect of chloroform or ether, may be controlled in the same way.

The frequency of the pulse-beat is increased by drinking hot water or tea, diminished by drinking these cold. Adding a warm covering to the clothing of the body increases the pulse by about ten beats a minute. Mental activity diminishes it more or less.

Ozokerite, or natural mineral wax, is found in large quantities in Utah. It is air, water, and acid proof, and can be used for imparting these qualities to other substances. It can also be adapted as the base of a cheap and hard paving material, and for coating posts to prevent decay.

The average weight of a man is 130 lbs.; of a woman, 118 lbs. A man's average height is 5 feet 9 inches; and a woman's, 5 feet 4 inches. In a new-born child, the pulse beats number 150 in a minute; at one year old, 110; at two, 95; from seven to fourteen, 85; in the adult, man, 72; and in woman, 80.

In Mott Street, New York, there is a large tenement house, inhabited chiefly by Roumanians and Poles, and containing a population of 478 persons. The death-rate in it is at the rate of 42 per thousand, while that of the city as a whole is 25 per thousand. Of the deaths 62 per cent. were children under five years of age. These large flats are often ill ventilated, and infection of all kinds spreads rapidly from one domicile to another.

During the American civil war village communities were often unable to obtain those comforts which have come to be necessaries of civilised life, and inventive minds turned themselves to improvising substitutes for tea, coffee, medicine, and the like. A recent writer tells how good they then thought coffee made of roasted hominy; sage, mint, and sassafras tea; and wild honey instead of sugar. Willow-bark tea was a successful substitute for quinine, and a mixture of lard and red pepper formed an admirable counter-irritant, especially efficacious in rheumatism.

It is not generally known that John Wesley was something of a physician, though his medical creed is founded more on theology than physiology. In a volume which he published in 1747, and which went through twenty-two editions in forty years, he states his views on the subject. He holds that the great antidote to many diseases is contained in the curse: "In the sweat of thy brow shalt thou eat bread; for the power of exercise, both to preserve and to restore health, is greater than can be conceived." He holds, however, that all medical treatment is, or ought to be, empirical; and decries scientific study. The prescriptions he gives are haphazard enough; here are a few examples: For a dropsy, eat a crust of bread every morning, or be electrified. For deafness, put a little salt in the ear. For a cancer of the mouth or throat, blow in the ashes of scarlet cloth. For a consumption, eat cow-heel soup, and every morning cut up a turf of fresh earth, and breathe into a hole for a quarter of an hour. And so forth. It is a pity that such a man as Wesley should have meddled in matters of which he had no knowledge.

